# Classification and Identification of S Haplotypes in Radish Based on *SRK* Kinase Domain Sequence Analysis

**DOI:** 10.3390/plants11172304

**Published:** 2022-09-02

**Authors:** Meng Ni, Xiaofang Yi, Qin Wang, Juan Wang, Shuang Wang, Liwang Liu, Liang Xu, Yan Wang

**Affiliations:** State Key Laboratory of Crop Genetics and Germplasm Enhancement/Key Laboratory of Biology and Germplasm Enhancement of Horticultural Crop in East China, Ministry of Agriculture and Rural Affairs, College of Horticulture, Nanjing Agricultural University, Nanjing 210095, China

**Keywords:** radish, self-incompatibility, *SRK* gene, S haplotype, PCR-RFLP

## Abstract

Radish is a typical self-incompatible crop. The rapid and accurate identification of S haplotypes can circumvent the blindness of the hybrid combination process, which is critical in radish heterosis utilization and the breeding of new varieties. In this study, based on the gene sequence which encodes the *S*-locus receptor kinase (SRK) of radish, and the polymerase chain reaction-restriction fragment length polymorphism (PCR-RFLP) analysis, the S haplotypes were identified among 79 cultivated radish genotypes. The PCR results indicated that 79 radish genotypes could be divided into 48 Class I, 13 Class II, and 17 Class I/II S haplotypes. Sequence alignment confirmed that the Class I materials contained 19 S haplotypes, of which three haplotypes (‘NAU-S53’, ‘NAU-S54’ and ‘NAU-S55’) were identified for the first time in radish. After digestion using the *Hin*f I restriction endonuclease, the *SRK* domain of DNA fragments of different genotypes showed high polymorphism. Homozygous materials S haplotypes could be quickly distinguished by the differences in the digested bands. Molecular identification of the S haplotype was highly consistent with the field pollination and pollen tube germination results. These results would provide an important approach for the rapid identification of radish S haplotypes and the efficient utilization of self-incompatibility in heterosis breeding.

## 1. Introduction

Radish (*Raphanus sativus* L.) is an economically important root vegetable crop belonging to the Brassicaceae family. As a self-incompatibility (SI) plant, radish exhibits obvious heterosis in hybrid seeds production. The SI is an important mechanism that prevents self-fertilization and maintains genetic diversity in flowering plants. Based on the genetic mechanism controlling the SI phenotype of the pollen, the SI systems are generally classified into Gametophytic SI (GSI) and Sporophytic SI (SSI). The pollen phenotype of GSI systems is conferred by the S genotypes of haploid pollen, while the phenotype of pollen grains in plants with SSI is determined by the S genotype of the diploid parent that produces pollen [1,2]. The SSI is controlled by one highly polymorphic *S*-locus containing three tightly linked genes such as the *S*-locus receptor kinase (SRK), *S*-locus glycoprotein (SLG) and *S*-locus cysteine rich (SCR), which has been well described in Brassica crops such as *B. rapa* [3] and *B. oleracea* [4]. Among them, *SRK* localized in the stigmatic papilla cells as the female determinant, and the pollen coat protein *SCR/SP11* controls the pollen determinant of SSI. On the basis of the SI phenotype and the sequence similarity of the S alleles, the S haplotypes in Brassica have been categorized into Class I and Class II. Class I haplotypes have a strong self-incompatible phenotypic effect and are generally considered dominant or co-dominant with other S haplotypes [5,6].

The SI system has been extensively used in F_1_ hybrid breeding of radish and other Brassica species, which has advantages including high efficiency and easy short-period breeding [7,8]. However, F_1_ seeds cannot be successfully produced at the anthesis stage if the parents share the same S-haplotype [6]. The rapid and accurate identification of S haplotypes can circumvent the blindness of the hybrid combination process, which is critical in radish heterosis utilization and the breeding of new varieties. In early studies, compatibility index analysis, pollen tube observation [9], fluorescence analysis [9,10] and isoelectric focusing gel examination [11] were often employed to conduct the S haplotype identification in Brassicaceae crops such as *B. oleracea* and *R. sativus*, however, it was difficult to widely apply for time consuming, labor costly and [12] complex operation. Polymerase chain reaction-restriction fragment length polymorphism (PCR-RFLP) has been successfully implemented to identify S haplotypes in *B. rapa* [13], *B. oleracea* [14], and *R. sativus* [15,16], but it has some drawbacks and is limited by whether the material is homozygous in the application.

With the increasing report about S locus gene sequences information, the polymorphism analysis of *SRK* or *SLG* gene sequences was developed into a new approach for accurate and rapid identification of S haplotype in many crops such as *R. sativus* [6] and Chinese cabbage [17]. In addition, alignment analysis of the only female determinant *SRK* has been reported to be reliable as a method for S haplotype identification, and has been used for the identification of S haplotypes of breeding lines in broccoli and cabbage [18,19]. Previous studies have shown that *S*-locus gene sequences are not conserved among different S haplotypes [20,21]. The identification of the radish S haplotype focused on the gene sequence of the S locus and was carried out by different teams around the world [7,15,22,23,24,25,26,27,28,29,30]. However, various groups use their own naming scheme for S haplotypes, which result in confusion of S haplotype names and limit the application of self-incompatibility of radish in heterosis breeding. Therefore, the unified naming of S haplotypes and the establishment of standard test lines are necessary for rapid and accurate identification of radish haplotypes.

In the present study, we firstly sorted out and named all of the currently published radish S haplotypes. Following, the S haplotypes of 79 cultivated radish genotypes were identified based on the gene sequence of *SRK* analysis, and also the PCR-RFLP marker was developed to rapidly classify the radish Class I S haplotype. In addition, the artificial pollination and pollen tube observation experiments were utilized to verify the accuracy of molecular identification. These results would provide an important theoretical basis for efficient utilization of self-incompatibility in heterosis breeding.

## 2. Results

### 2.1. Comparison and Unified Nomenclature of Published S haplotype in Radish (Raphanus sativus L.)

In previous reports, a total of 35 S haplotypes were found by Nishio [29] and Haseyama [30] in radish, but the results were incomplete. Therefore, comprehensive published information on radish S haplotypes [7,15,22,23,24,25,26,27,28,29,30] were collected, and their nucleotide and amino acid sequences were futher compared in this study (Table S1). It was found that the S haplotypes identified by the teams intersected with each other (Figure S1). After removing the redundant members based on BLAST analysis of the reported S gene nucleotide and amino acid sequence, 52 S haplotypes have been identified in radish, which were numbered as ‘NAU-S1’-‘NAU-S52’ (Table 1).

### 2.2. SRK Kinase Domain-Based Classification and Identification of S haplotypes

**Figure 1:**
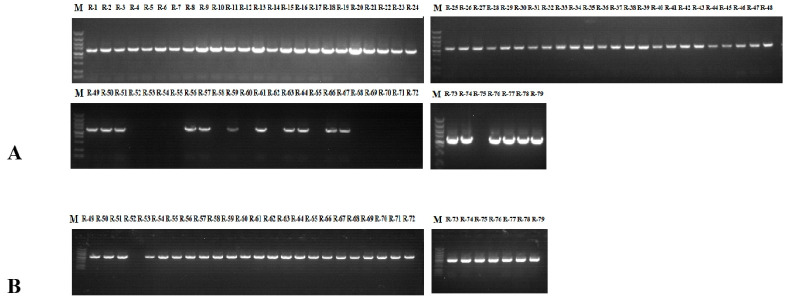
PCR amplification of *SRK* kinase domain in radish. (**A**) PCR amplification results of KD(I)-F/R in NAU-R1-NAU-R79; (**B**) PCR amplification results of KD4/KD7 in NAU-R49-NAU-R79.

Class Ⅰ and Class Ⅱ primers were designed based on the sequence of the *SRK* kinase region, which can be applied to classify the compatibility of different genotypes. Class Ⅰ exhibits strong self-incompatibility, while Class Ⅱ exhibits weak self-incompatibility. Incompatibility Classes I and II show a dominant relationship [31,32]. The primers KD (I)-F/R and KD4/KD7 can respectively generate 1200 (Figure 1A) and 1000 bp (Figure 1B) bands among the radish materials. PCR results shown that the 79 radish genotypes could be divided into Class I (48), Class II (13) and Class I/II (17) groups. Homozygous Class I and II S haplotype materials are found in 60.76% and 16.46% of radish, respectively. Strong self-incompatibility occurs more frequently (Table 2). 

The types and frequencies of S haplotypes in the tested radish materials were analyzed according to the S haplotype system established in this study. The 48 Class I materials contained 19 sequence types, of which 16 indicated high similarities to known S haplotypes. Among them, the nucleotide sequences of eight lines were highly similar to Okamoto (S22, S7), Lim (S16), and Kim D (S13) and were identified as ‘NAU-S16’, with a frequency of up to 16.67%. Likewise, seven lines were ‘NAU-S25’, with a frequency of 14.58% as well as five lines were ‘NAU-S17’, with a frequency of 10.41%. There were four materials of ‘NAU-S51’ type, with a frequency of 8.33%. The ‘NAU-S04’ and ‘NAU-S44’ types comprised three materials, and the frequency was 6.25%. The ‘NAU-S02’ and ‘NAU-S14’ types appeared twice, and the other S haplotypes appeared only once in the tested materials (Table 3). The predicted amino acid sequence was used in a protein BLAST search. The *SRK* sequence information of NAU-Rs46, NAU-Rs47, and NAU-Rs48, has not been found in the NCBI database. It was thus preliminarily inferred that there were three new S haplotypes, which were respectively named as ‘NAU-S53’, ‘NAU-S54’, and ‘NAU-S55’ (The nucleotide sequences of new S haplotypes are listed in Table S2).

The S haplotypes of the 13 Class II radish genotypes identified were focused on four types including ‘NAU-S38’, ‘NAU-S39’, ‘NAU-S43’, and ‘NAU-S52’ (Table 4). In addition, the ClassI/II *SRK* gene could be simultaneously amplified by the primer of KD (D)-F/R and KD4/KD7 among 17 materials which maybe heterozygous at the S locus (Table 5).

### 2.3. PCR-RFLP Analysis of SRK Alleles

**Figure 2:**
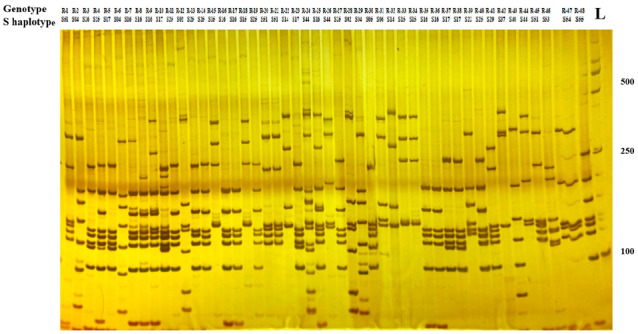
Polyacrylamide gel electrophoresis of PCR products after cleavage with *Hin*f I. L: 2000-bp ladders.

Compared with the sequence alignment analysis, the utilization of PCR-RFLP technology to identify the S haplotype of radish materials does not require sequencing and has the advantages of a faster and higher identification efficiency and lower cost. To identify and classify the S haplotypes from genotypes with SI phenotypes, the PCR reaction was performed with the Class I *SRK* specific primer, and then digestion of the PCR products with *Hin*f I restriction endonucleases and subsequent polyacrylamide gel electrophoresis revealed polymorphism of the amplified DNA fragments. There were 19 types of electrophoretic profiles found in 48 genotypes (Figure 2). The size of the electronic restriction fragments of the PCR product is attached in Table S3. The PCR-RFLP result was the same as the nucleotide sequence analysis. All the different S genotypes showed different electrophoretic profiles, while lines with the same S genotype had the same electrophoretic profiles. These results showed that the self-incompatibility and S haplotype of radish homozygous genotypes could be quickly discovered through employing PCR-RFLP markers to analyze *SRK* gene polymorphisms.

### 2.4. Pollen Germination and Tube Growth

The germination and growth of pollen tube would be inhibited on the stigma of those self-incompatible lines belonging to same S haplotypes. Consequently, fertilization does not occur and no seeds are produced. To verify the molecular identification results of the S haplotype, different S haplotype materials were prepared for pollination, and pollen tube germination after hybridization/self-pollination at the flowering stage was analyzed. 

**Figure 3:**
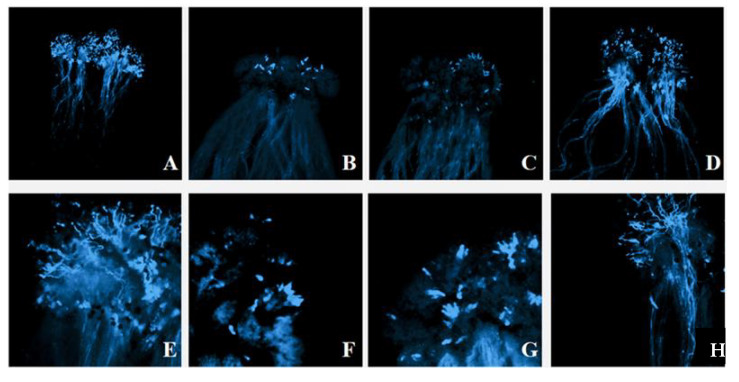
The germination of pollen tubes of different pollination combinations. (**A**). NAU-Rs4 (‘NAU-S25’) Bud pollination⊗ (4×); (**B**). NAU-Rs4 (‘NAU-S25’) Flower pollination⊗ (4×); (**C**). NAU-Rs4 × NAU-Rs40 (‘NAU-S25’) (4×); (**D**). NAU-Rs4 × NAU-Rs32 (‘NAU-S25’ × ‘NAU-S14’) (4×); (**E**). NAU-Rs4 (‘NAU-S25’) Bud pollination⊗ (10×); (**F**). NAU-Rs4 (‘NAU-S25’) Flower pollination⊗ (10×); (**G**). NAU-Rs4 × NAU-Rs40 (‘NAU-S25’) (10×); (**H**). NAU-Rs4 × NAU-Rs32 (‘NAU-S25’ × ‘NAU-S14’) (10×).

The strong self-incompatibility radish line ‘NAU-Rs4’ was employed as an example to explore the behavior of pollen tube in the stigma, and the germination of self-pollinated pollen tubes at the bud stage was normal (Figure 3A,E). When the same material was self-pollinated during flowering, it was obvious that pollen grains germinated less at the stigma and failed to produce pollen tubes that extended to the style (Figure 3B,F). This was similar to the result after cross-pollination of the same S haplotype material NAU-Rs4 × NAU-Rs40 (‘NAU-S25’), which induced the callose response (Figure 3C,G). In contrast, different S haplotype materials ‘NAU-Rs4’ × ’NAU-Rs32’ (‘NAU-S25’ × ‘NAU-S14’) were cross-pollinated at the flowering stage, which resulted in a large number of pollen grains germinating, and pollen tube elongation was observed (Figure 3D,H). These results of the fluorescence microscope observation of pollen tube germination verified the accuracy of S haplotypes identification.

### 2.5. Compatibility Index Analysis

To verify the molecular identification results of the S haplotype, different cross combinations were conducted, and the compatibility was analyzed after pollination. 

Cross combinations included the same S haplotype material: NAU-Rs5 × NAU-Rs38 (‘NAU-S17’), NAU-Rs44 × NAU-Rs24 (‘NAU-S44’), NAU-Rs7 × NAU-Rs9 (‘NAU-S16’), NAU-Rs7 × NAU-Rs16 (‘NAU-S16’), NAU-Rs4 × NAU-Rs40 (‘NAU-S25’, orthogonal), NAU-Rs40 × NAU-Rs4 (‘NAU-S25’, reverse cross); and different S haplotype material: NAU-Rs1 × NAU-Rs40, NAU-Rs44 × NAU-Rs30, NAU-Rs34 × NAU-Rs40, NAU-Rs7 × NAU-Rs18, and NAU-Rs7 × NAU-Rs1. At the same time, four materials of NAU-Rs1 (‘NAU-S51’), NAU-Rs45 (‘NAU-S05’), NAU-Rs33 (‘NAU-S15’), and NAU-Rs30 (‘NAU-S51’) were used as controls during the flowering stage. 

**Figure 4:**
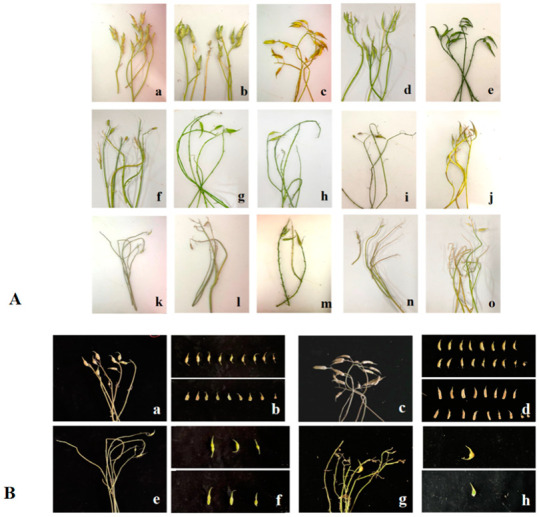
The pod setting and seed setting of different pollination combinations. (**A**). (**a**). NAU-Rs1 × NAU-Rs40 (‘NAU-S51’ × ‘NAU-S25’); (**b**). NAU-Rs34 × NAU-Rs40 (‘NAU-S15’ × ‘NAU-S25’); (**c**). NAU-Rs7 × NAU-Rs18 (‘NAU-S16’ × ‘NAU-S15’); (**d**). NAU-Rs44 × NAU-Rs30 (‘NAU-S44’ × ‘NAU-S05’); (**e**). NAU-Rs7 × NAU-Rs1 (‘NAU-S16’ × ‘NAU-S51’); (**f**). NAU-Rs5 × NAU-Rs38 (‘NAU-S17’); (**g**). NAU-Rs40 × NAU-Rs4 (‘NAU-S25’); (**h**). NAU-Rs7 × NAU-Rs9 (‘NAU-S16’); (**i**). NAU-Rs7 × NAU-Rs16 (‘NAU-S16’); (**j**). NAU-Rs44 × NAU-Rs24 (‘NAU-S44’); (**k**). NAU-Rs4 × NAU-Rs40 (‘NAU-S25’); (**l**). NAU-Rs1⊗ (‘NAU-S51’); (**m**). NAU-Rs30⊗ (‘NAU-S05’); (**n**). NAU-Rs3⊗ (‘NAU-S15’); (**o**). NAU-Rs45⊗ (‘NAU-S51’). (**B**). (**a**,**b**). NAU-Rs7 × NAU-Rs18 (‘NAU-S16’ × ‘NAU-S15’); (**c**,**d**). NAU-Rs7 × NAU-Rs1 (‘NAU-S16’ × ‘NAU-S51’); (**e**,**f**). NAU-Rs4 × NAU-Rs40 (‘NAU-S25’); (**g**,**h**). NAU-Rs7 × NAU-Rs16 (‘NAU-S16’).

The seed production of the F_1_ generation depends on whether the parents have the same S haplotype. After 40–50 days of pollination, the hybridization of the same S haplotype was almost sterile. The compatibility index was less than 1, and the reciprocal cross results were consistent, which was similar to the self-pollinating result of SI material in the flowering period. In contrast, the compatibility indexes of different S haplotypes materials are all larger than two, showing compatibility (Table 6, Figure 4). The results of self- and cross-pollination tests were consistent with the prediction of S haplotypes by PCR analysis.

## 3. Discussion

As a self-incompatibility (SI) plant, radish exhibits high heterosis in hybrid seed production. However, F_1_ seeds cannot be produced at the anthesis stage because the parents share the same S haplotype [6]. To ensure rational hybridization and guarantee the purity and yield of the hybrid, it is necessary to rapidly and accurately identify the S haplotype of the radish hybrid parent.

The unified naming of S haplotypes and the establishment of standard test lines are necessary for rapid and accurate identification of radish haplotypes. Haseyama [30] determined the reported *SRK*, *SLG*, and *SCR/SP11* gene sequences and found that there were 35 S haplotypes in radish, of which there were 26 ones in South Korea radish and 24 ones in Japanese radish. A BLAST analysis revealed that 15 S haplotypes are widespread in Japanese and South Korean radish. However, the results failed to cover all current radish haplotype information. Therefore, in this study, all published S gene nucleotide sequences were collected for BLAST alignment. On the basis research of Haseyama [30], the S haplotype reported by Kim [28], Wang [6] and other teams was added. It was concluded that a total of 52 S haplotypes were reported in radish and they were uniformly named ‘NAU-S1’-‘NAU-S52’ (Table 1). Exchanges of plant materials between researchers and breeders and the establishment of a unified nomenclature of S haplotypes are necessary to avoid confusion regarding the identity of S haplotypes in radish. The S haplotype is an important agronomic trait of cruciferous crops that varies greatly among different species. Thus far, more than 50 S haplotypes have been found in *B. oleracea* crops [5], which is comparable to the number of radish S haplotypes determined in the present study. And more than 100 S haplotypes have been identified in Brassica [32,33,34]. There are many *SRK* and *SP11/SCR* alleles having highly similar sequences between *B. oleracea* and *B. rapa.* Similar interspecific pairs of S haplotypes also exist in radish. Due to the large number of S haplotypes, traditional pollination methods are complicated to distinguish S haplotypes [27]. Establishing an efficient and simple S haplotype identification system is important for the breeding work. 

To establish a reliable S haplotyping system in radish, we designed specific primers to analyze the *SRK* kinase domain sequence. The PCR results indicated that 79 radish genotypes could be divided into 48 Class I, 13 Class II, and 17 Class I/II S genotypes. Sequence alignment confirmed that the Class I materials contained 19 S haplotypes. Among them, the S sequences of the three materials were not registered in NCBI, and hence, there were new members identified for the first time in radish, named ‘NAU-S53’, ‘NAU-S54’, and ‘NAU-S55’. In general, Class I S haplotypes predominate over Class II S haplotypes. In the study, the S haplotypes of Class II materials were only concentrated in four types, such as ‘NAU-S38’ and the types and numbers are significantly less than those of Class I S haplotypes. It is convincing that strong self-incompatibility lines are more common. In previous reports, this phenomenon was also found in *B. campestris* [35] and *B. pekinensis Rupr* [36,37,38]. Directional selection, either natural or through breeding, increases the frequency of favorable S alleles resulting in the differences of S haplotype frequencies. 

The publication of S locus gene sequence details has enabled the identification of S haplotypes based on gene sequences and BLAST analyses. However, in breeding practice, when conducting large-scale screening of parents to prepare hybrid combinations, all parent materials need to be sequenced. This entails low throughput and high cost, which are unsuitable for batch identification of S haplotypes. In contrast, PCR-RFLP markers have the characteristics of high polymorphism, good reproducibility and codominance, which are easily used to develop efficient, simple and practical molecular markers [39], and successfully detect S haplotypes in crops such as *B. campestris* and Chinese cabbage, as well as fruit plants such as apple and pear [14,40,41,42]. The S haplotype of 48 Class I materials was identified using the PCR-RFLP technique here, and their PCR products showed a polymorphism depending on their genotypes. Therefore, the PCR-RFLP analysis of *S*-locus allele is adequate for the S haplotypes identification.

The PCR-RFLP method has proven useful for the identification of S alleles in genotypes and listing S haplotypes in radish [16]. Of course, the limitation of this method is that some S haplotypes have a significant degree of sequence similarity or the same restriction site, making the PCR-RFLP approach ineffective. Furthermore, it is difficult to identify individual haplotypes for heterozygotes or new S haplotypes [36,37]. Thus, for materials with complex bands are difficult to distinguish accurately. PCR-RFLP analysis, combined with the cloning analysis of the S locus gene, permits rapid and efficient identification of the radish S haplotype.

In addition, the compatibility relationships in pollen between same S haplotypes of radish by pollination tests and aniline blue tests were analyzed. The results of molecular identification of the S haplotype were highly consistent with the field pollination and pollen tube germination. It will become an essential tool based on the combination of radish *SRK* gene sequence analysis and the PCR-RFLP in radish breeding, which would provide an important theoretical basis for efficient utilization of self-incompatibility in heterosis breeding.

## 4. Materials and Methods

### 4.1. Plant Materials

The 79 cultivated radish genotypes were provided by the Radish Genetics and Breeding Laboratory, School of Horticulture, Nanjing Agricultural University (Table 7). Pollination experiments were conducted in 2019–2021 at Jiangpu Horticultural Experimental Station and Baima Experimental Base of Nanjing Agricultural University.

### 4.2. Amplification and Sequencing of the PCR Products

#### 4.2.1. Extraction of Genomic DNA and Amplification of the *SRK* Gene

Total genomic DNA was extracted from the seedling leaves of each genotype using the modified CTAB (Cetyltrimethylammonium Bromide) method. Degenerate primers were designed based on the nucleotide sequence (exons 4–7) of the radish *SRK* gene (kinase domain) published by Lim [16], Okamoto [23] et al. The sequences encoding the kinase domains of Class I and Class II S haplotype *SRK* were amplified with KD (I)-F/R and KD4/KD7. The primer sequences were shown in Table 8. 

PCR amplification was performed in a 20 μL reaction mixture containing 0.1 μg template, 1 μL forward primer (10 μM), 1 μL reverse primer (10 μM), and 10 μL polymerase mix (2 × Taq Master Mix, Vazyme Biotech Co., Ltd., Nanjing, China). PCR amplification consisted of an initial denaturation step at 94 °C for 5 min, 35 cycles of 95 °C for 30 s, 59 °C for 30 s, and 72 °C for 1 min 40 s, and a final 10 min extension at 72 °C. 

#### 4.2.2. Determination of Nucleotide Sequences

The PCR products were visualized on 1.2% agarose gel. The FastPure Plant DNA Isolation Mini Kit^®^ (Vazyme Biotech Co., Ltd., Nanjing, China) was used for product purification. Then, it was ligated with the cloning vector pMD19-T and transformed into *E. coli* (Escherichia coli) competent DH5α. Sequencing reactions were performed by Spokane Biotech Co., Ltd. (Nanjing, China). The obtained nucleotide sequences of the *SRK* gene of the respective cross lines were compared in the NCBI (National Center for Biotechnology Information) database to determine the corresponding haplotype. 

### 4.3. PCR-RFLP Analysis

DNA fragments corresponding to the *SRK* kinase domain were amplified by PCR with a Class I specific primer pair, KD (I)-F and KD (I)-R. The reaction conditions and system were identical to those described in Section 4.2. The PCR products were subjected to restriction digestion using *Hin*f I restriction enzyme at 37 °C for 1 h, and 65 °C for 20 min. The digested product was electrophoresed on a 6% non-denaturing polyacrylamide gel at a constant voltage 120 V for 2 h, and DNA bands were detected by silver staining. 

### 4.4. Pollination Tests

The traditional compatibility index method was used to verify the molecular identification results of the S haplotype. Based on the results of the *SRK* gene sequence comparison, artificial cross-pollination was carried out within the same haplotype and among different haplotypes at the flowering stage to determine whether the materials belonged to the same S haplotype according to the compatibility index and pod setting rate among different hybrid combinations.

Pod setting rate = number of pods/number of pollinated flowers × 100%.

Compatibility index = number of seeds/number of pollinated flowers.

In the field pollination statistics of radish, the compatibility index less than 0.5 indicates strong self-incompatibility, a value greater than 2.0 indicates compatibility, and compatibility index from 0.5 to 2.0 indicates weak self-incompatibility [7].

### 4.5. Aniline Blue Assays

Aniline blue assays were performed as previously described. Based on the molecular identification results of the S haplotype, the pistils after hybridization with the same or different S haplotypes were fixed in FAA (Formalin-Aceto-Alcohol) fixative (50% Ethanol:Glacial Acetic Acid:Ormaldehyde = V18:V1:V1) at least 4 h and then transferred to 10 M NaOH at 42 °C for 0.5 h. The pistils were washed with distilled water and stained with 0.1% basic aniline blue (1% K_3_PO_3_, PH = 11) [43,44]. The stained samples were mounted in 70% glycerol and the growth of pollen tubes in the styles was observed under a BX53^®^ Olympus fluorescence microscope (Olympus, Tokyo, Japan).

## Figures and Tables

**Table 1:** Summary of S haplotypes in radish.

S Haplotyes (Renumbered in This Study)	S Haplotypes (Okamoto) [23]	S Haplotypes (Lim) [16]	S Haplotypes (Kim D) [26,27,28]	S Haplotypes (Other Group)	Similarity Okamoto vs. Lim	Similarity Lim vs. Kim D (or Other Group)
NAU-S1	S1	S6	S31		99.8%	100%
NAU-S2	S2	S2	S2		100%	100%
NAU-S3	S3	S12			99.9%	
NAU-S4	S4	S7	S6		100%	100%
NAU-S5	S5					
NAU-S6	S6	S18	S15		100%	100%
NAU-S7	S8					
NAU-S8	S9	S21		S4/S5 (*SP11-4/5*) (Kim H, 2003)	99.3%	98.7%
NAU-S9	S11					
NAU-S10	S14					
NAU-S11	S15			S2 (*SP11-2*) (Kim H, 2003)	100%	
NAU-S12	S17	S9	S5		99.7%	100%
NAU-S13	S18					
NAU-S14	S19	S8	S7 (S30)		100%	100%
NAU-S15	S21					
NAU-S16	S22 (S7)	S16	S13		100%	99.1%
NAU-S17	S23	S20	S16		99.9%	100%
NAU-S18	S25	S17			99.9%	
NAU-S19	S26	S4		S1 (*SP11-1*) (Kim H, 2003)	100%	100%
NAU-S20	S28					
NAU-S21	S29	S26		S3 (*SP11-3*) (Kim H, 2003)	99.4%	100%
NAU-S22	S30	S1	S1		100%	100%
NAU-S23	S31					
NAU-S24		S5				
NAU-S25		S10	S14			99.73%
NAU-S26		S11	S9 (S27)	S38 [6] DQ984139 [7]		100%
NAU-S27		S15	S12			100%
NAU-S28		S22				
NAU-S29		S23	S19			100%
NAU-S30		S24				
NAU-S31		S27	S24			99.8%
NAU-S32		S29				
NAU-S33		S30				
NAU-S34		S31	S29			100%
NAU-S35			S4			
NAU-S36			S8			
NAU-S37			S10			
NAU-S38			S11			
NAU-S39			S17			
NAU-S40			S18			
NAU-S41			S20			
NAU-S42			S21			
NAU-S43			S22			
NAU-S44			S23			
NAU-S45			S25	S39 [6]		100%
NAU-S46			S28			
NAU-S47				S201 (Niikura, 1997)		
NAU-S48				EF056499 [7]		
NAU-S49				GQ121139 [22]		
NAU-S50				S40 [6]		
NAU-S51				S48 (Kim S, 2021)		
NAU-S52				S6 [25]		

**Table 2:** The classification of S haplotypes in radish.

Class of S Haplotypes	Genotype	Frequency (%)
Class I	NAU-Rs1, NAU-Rs2, NAU-Rs3, NAU-Rs4, NAU-Rs5, NAU-Rs6, NAU-Rs7, NAU-Rs8, NAU-Rs9, NAU-Rs10, NAU-Rs11, NAU-Rs12, NAU-Rs13, NAU-Rs14, NAU-Rs15, NAU-Rs16, NAU-Rs17, NAU-Rs18, NAU-Rs19, NAU-Rs20, NAU-Rs21, NAU-Rs22, NAU-Rs23, NAU-Rs24, NAU-Rs25, NAU-Rs26, NAU-Rs27, NAU-Rs28, NAU-Rs29, NAU-Rs30, NAU-Rs31, NAU-Rs32, NAU-Rs33, NAU-Rs34, NAU-Rs35, NAU-Rs36, NAU-Rs37, NAU-Rs38, NAU-Rs39, NAU-Rs40, NAU-Rs41, NAU-Rs42, NAU-Rs43, NAU-Rs44, NAU-Rs45, NAU-Rs46, NAU-Rs47, NAU-Rs48	60. 76
Class II	NAU-Rs53, NAU-Rs54, NAU-Rs55, NAU-Rs58, NAU-Rs60, NAU-Rs62, NAU-Rs65, NAU-Rs68, NAU-Rs69, NAU-Rs70, NAU-Rs71, NAU-Rs72, NAU-Rs75	16. 46
Class I/Class II	NAU-Rs49, NAU-Rs50, NAU-Rs51, NAU-Rs56, NAU-Rs57, NAU-Rs59, NAU-Rs61, NAU-Rs63, NAU-Rs64, NAU-Rs66, NAU-Rs67, NAU-Rs73, NAU-Rs74, NAU-Rs76, NAU-Rs77, NAU-Rs78, NAU-Rs79	21. 52

**Table 3:** S haplotypes frequencies among 48 Class I radish materials.

S Haplotype	Number of Occurrences	Frequency (%)
NAU-S16	8	16. 67
NAU-S25	7	14. 58
NAU-S17	5	10. 41
NAU-S15/NAU-S51	4	8. 33
NAU-S04/NAU-S44	3	6. 25
NAU-S02/NAU-S14	2	4. 17
NAU-S05/NAU-S06/NAU-S22/NAU-S26/NAU-S29/NAU-S37/NAU-S40NAU-S53/NAU-S54/NAU-S55	1	2. 08

**Table 4:** S haplotypes frequencies among 13 Class II radish materials.

S Haplotype	Genotype	Number of Occurrences	Frequency (%)
NAU-S39	NAU-Rs53, NAU-Rs55, NAU-Rs58, NAU-Rs70, NAU-Rs72	5	38.46
NAU-S52	NAU-Rs60, NAU-Rs65, NAU-Rs69, NAU-Rs75	4	30.77
NAU-S43	NAU-Rs54, NAU-Rs68, NAU-Rs71	3	23.08
NAU-S38	NAU-Rs62	1	7.69

**Table 5:** Distribution of S haplotype in 17 Class I/II radish materials.

Genotype	S Haplotype (Class I)	S Haplotype (Class II)
NAU-Rs49	NAU-S17	NAU-S39
NAU-Rs50	NAU-S17	NAU-S43
NAU-Rs51	NAU-S17	NAU-S43
NAU-Rs56	NAU-S26	NAU-S43
NAU-Rs57	NAU-S14	NAU-S43
NAU-Rs59	NAU-S17	NAU-S52
NAU-Rs61	NAU-S17	NAU-S52
NAU-Rs63	NAU-S26	NAU-S39
NAU-Rs64	NAU-S17	NAU-S39
NAU-Rs66	NAU-S26	NAU-S38
NAU-Rs67	NAU-S25	NAU-S52
NAU-Rs73	NAU-S26	NAU-S38
NAU-Rs74	NAU-S26	NAU-S52
NAU-Rs76	NAU-S17	NAU-S52
NAU-Rs77	NAU-S55	NAU-S39
NAU-Rs78	NAU-S26	NAU-S39
NAU-Rs79	NAU-S17	NAU-S52

**Table 6:** Compatibility index and podding rate of different pollination combinations.

Pollination Combinations	Compatibility Index	Podding Rate (%)	Compatibility	S Haplotype
NAU-Rs5 × NAU-Rs38	0.88	27.57	Weak Incompatibility	NAU-S17
NAU-Rs44 × NAU-Rs24	0.52	24.09	Weak Incompatibility	NAU-S44
NAU-Rs7 × NAU-Rs9	0.20	12.07	Incompatibility	NAU-S16
NAU-Rs7 × NAU-Rs16	0.17	12.09	Incompatibility	NAU-S16
NAU-Rs4 × NAU-Rs40	0.47	19.75	Incompatibility	NAU-S25
NAU-Rs40 × NAU-Rs4	0.37	21.79	Incompatibility	NAU-S25
NAU-Rs1 × NAU-Rs40	3.76	78.93	Compatibility	NAU-S51, NAU-S25
NAU-Rs44 × NAU-Rs30	3.08	74.64	Compatibility	NAU-S44, NAU-S05
NAU-Rs34 × NAU-Rs40	2.83	67.90	Compatibility	NAU-S15, NAU-S25
NAU-Rs7 × NAU-Rs18	1.85	72.98	Weak Incompatibility	NAU-S16, NAU-S15
NAU-Rs7 × NAU-Rs1	2.05	71.75	Compatibility	NAU-S16, NAU-S51
NAU-Rs1⊗	0.09	12.40	Self-incompatibility	NAU-S51
NAU-Rs30⊗	0.46	20.69	Self-incompatibility	NAU-S05
NAU-Rs33⊗	0.17	13.22	Self-incompatibility	NAU-S15
NAU-Rs45⊗	0.11	14.59	Self-incompatibility	NAU-S51

**Table 7:** Materials of radish used in this study.

Material Code	Color of Root Skin	Color of Fleshy Root	Fleshy Root Shape	Leaf Morphology	Source
NAU-Rs1	Green	Green	Cylindrical	Entire	Henan China
NAU-Rs2	Red	White	Spherical	Entire	Jiangsu China
NAU-Rs3	Green	Green	Cylindrical	Entire	Henan China
NAU-Rs4	Green	Green	Cylindrical	Lyrate	Jiangsu China
NAU-Rs5	Red	White	Spherical	Entire	Sichuan China
NAU-Rs6	White	White	Long and Tapered	Entire	Jiangsu China
NAU-Rs7	White	White	Long and Tapered	Entire	Beijing China
NAU-Rs8	White	White	Long and Tapered	Entire	Jiangsu China
NAU-Rs9	White	White	Long and Tapered	Lyrate	Jiangsu China
NAU-Rs10	Red	White	Cylindrical	Entire	Sichuan China
NAU-Rs11	White	White	Cylindrical	Lyrate	South Korea
NAU-Rs12	Red	White	Apically Bulbous	Lyrate	America
NAU-Rs13	White	White	Cylindrical	Lyrate	Jiangsu China
NAU-Rs14	Red	White	Elliptic	Lyrate	Jiangsu China
NAU-Rs15	Red	White	Elliptic	Lyrate	Jiangsu China
NAU-Rs16	White	White	Long and Tapered	Lyrate	South Korea
NAU-Rs17	White	White	Long and Tapered	Lyrate	South Korea
NAU-Rs18	White	White	Long and Tapered	Lyrate	Guangdong China
NAU-Rs19	Purple	White	Apically Bulbous	Lyrate	Wuhan China
NAU-Rs20	White	White	Long and Tapered	Lyrate	Jiangsu China
NAU-Rs21	Red	White	Long and Tapered	Entire	Jiangsu China
NAU-Rs22	White	White	Long and Tapered	Lyrate	South Korea
NAU-Rs23	Green	White	Apically Bulbous	Lyrate	Henan China
NAU-Rs24	Red	White	Elliptic	Entire	Jiangsu China
NAU-Rs25	White	White	Long and Tapered	Entire	South Korea
NAU-Rs26	White	White	Long and Tapered	Sinuate	Wuhan China
NAU-Rs27	Purple	White	Apically Bulbous	Lyrate	Xizang China
NAU-Rs28	White	White	Spherical	Lyrate	Jiangsu China
NAU-Rs29	Red	White	Elliptic	Entire	Jiangsu China
NAU-Rs30	White	White	Long and Tapered	Lyrate	Anhui China
NAU-Rs31	White	White	Apically Bulbous	Lyrate	Jiangsu China
NAU-Rs32	Red	White	Elliptic	Entire	Jiangsu China
NAU-Rs33	Green	Red	Spherical	Entire	Jiangsu China
NAU-Rs34	White	White	Cylindrical	Lyrate	South Korea
NAU-Rs35	White	White	Long and Tapered	Lyrate	Jiangsu China
NAU-Rs36	Yellow	White	Spherical	Lyrate	Jiangsu China
NAU-Rs37	Red	White	Cylindrical	Entire	Sichuan China
NAU-Rs38	White	White	Apically Bulbous	Entire	Jiangsu China
NAU-Rs39	Red	White	Cylindrical	Entire	Shandong China
NAU-Rs40	Green	Green	Long and Tapered	Lyrate	Shandong China
NAU-Rs41	Green	Green	Cylindrical	Lyrate	Jiangsu China
NAU-Rs42	White	White	Cylindrical	Lyrate	Jiangsu China
NAU-Rs43	Green	Green	Apically Bulbous	Lyrate	Henan China
NAU-Rs44	Red	White	Apically Bulbous	Entire	Jiangsu China
NAU-Rs45	White	White	Elliptic	Lyrate	Jiangsu China
NAU-Rs46	White	White	Spherical	Lyrate	Jiangsu China
NAU-Rs47	Red	White	Long and Tapered	Entire	Sichuan China
NAU-Rs48	Red	White	Cylindrical	Sinuate	Jiangsu China
NAU-Rs49	White	White	Long and Tapered	Entire	Yunnan China
NAU-Rs50	Red	White	Cylindrical	Sinuate	Jiangsu China
NAU-Rs51	Green	Green	Cylindrical	Entire	Shandong China
NAU-Rs52	Green	Red	Cylindrical	Entire	Beijing China
NAU-Rs53	White	White	Long and Tapered	Lyrate	Japan
NAU-Rs54	Red	White	Cylindrical	Sinuate	Sichuan China
NAU-Rs55	White	White	Cylindrical	Sinuate	Jiangsu China
NAU-Rs56	Red	White	Cylindrical	Sinuate	Jiangsu China
NAU-Rs57	White	White	Cylindrical	Sinuate	South Korea
NAU-Rs58	White	White	Long and Tapered	Sinuate	Jiangsu China
NAU-Rs59	White	White	Cylindrical	Entire	Japan
NAU-Rs60	White	White	Cylindrical	Entire	South Korea
NAU-Rs61	White	White	Cylindrical	Entire	Jiangsu China
NAU-Rs62	White	White	Long and Tapered	Entire	South Korea
NAU-Rs63	White	White	Cylindrical	Entire	Jiangsu China
NAU-Rs64	White	White	Cylindrical	Entire	Jiangsu China
NAU-Rs65	White	White	Long and Tapered	Entire	South Korea
NAU-Rs66	Red	White	Cylindrical	Entire	Jiangsu China
NAU-Rs67	White	White	Long and Tapered	Entire	Jiangsu China
NAU-Rs68	White	White	Long and Tapered	Entire	Jiangsu China
NAU-Rs69	Red	White	Cylindrical	Entire	Jiangsu China
NAU-Rs70	Green	Green	Cylindrical	Entire	Tianjin China
NAU-Rs71	Green	White	Cylindrical	Entire	Henan China
NAU-Rs72	Green	White	Cylindrical	Entire	Shandong China
NAU-Rs73	White	White	Long and Tapered	Entire	Jiangsu China
NAU-Rs74	Red	White	Cylindrical	Entire	Jiangsu China
NAU-Rs75	White	White	Long and Tapered	Entire	Shandong China
NAU-Rs76	Red	White	Long and Tapered	Entire	Jiangsu China
NAU-Rs77	White	White	Long and Tapered	Lyrate	Jiangsu China
NAU-Rs78	White	White	Long and Tapered	Entire	Beijing China
NAU-Rs79	White	White	Long and Tapered	Entire	Jiangsu China

**Table 8:** Specific primers of *SRK* gene.

Primer Name	Class	Gene Regions	Primer Sequence (5′ to 3′)	Tm/°C
KD (I)-F	Class I	*SRK* (exons 4–7)	GAACTTCCATTGATAGAGTTRG	58.5
KD (I)-R			TTRGGCTKAGGAATCKCT	
KD4 [42]	Class II		GAGGGCGAGAAAGATCTTAATT	59.5
KD7 [42]			AAGACGATCATATTACCGAGC	

## Data Availability

The data presented in this study are available on request from the corresponding author.

## Supplementary Materials

The following supporting information can be downloaded at: https://www.mdpi.com/article/10.3390/plants11172304/s1, **Table S1**: Summary of the publicly published radish S haplotypes; Table S2: Novel S haplotype gene sequence; Table S3: Size of restriction fragment of PCR product of Class I *SRK* gene; Figure S1: Venn diagram of radish S haplotypes determined by groups.
