# Supplementary material for: Classification and Identification of S Haplotypes in Radish Based on SRK Kinase Domain Sequence Analysis
Source: Plants (Basel). 2022 Sep 2;11(17):2304. doi: 10.3390/plants11172304 (PMC9459979; doi:10.3390/plants11172304)
Supplement: Supplementary file 1 [file plants-11-02304-s001.zip › Supplement 2022.9.1/Figure S1.Venn diagram of radish S haplotypes determined by groups.pdf]

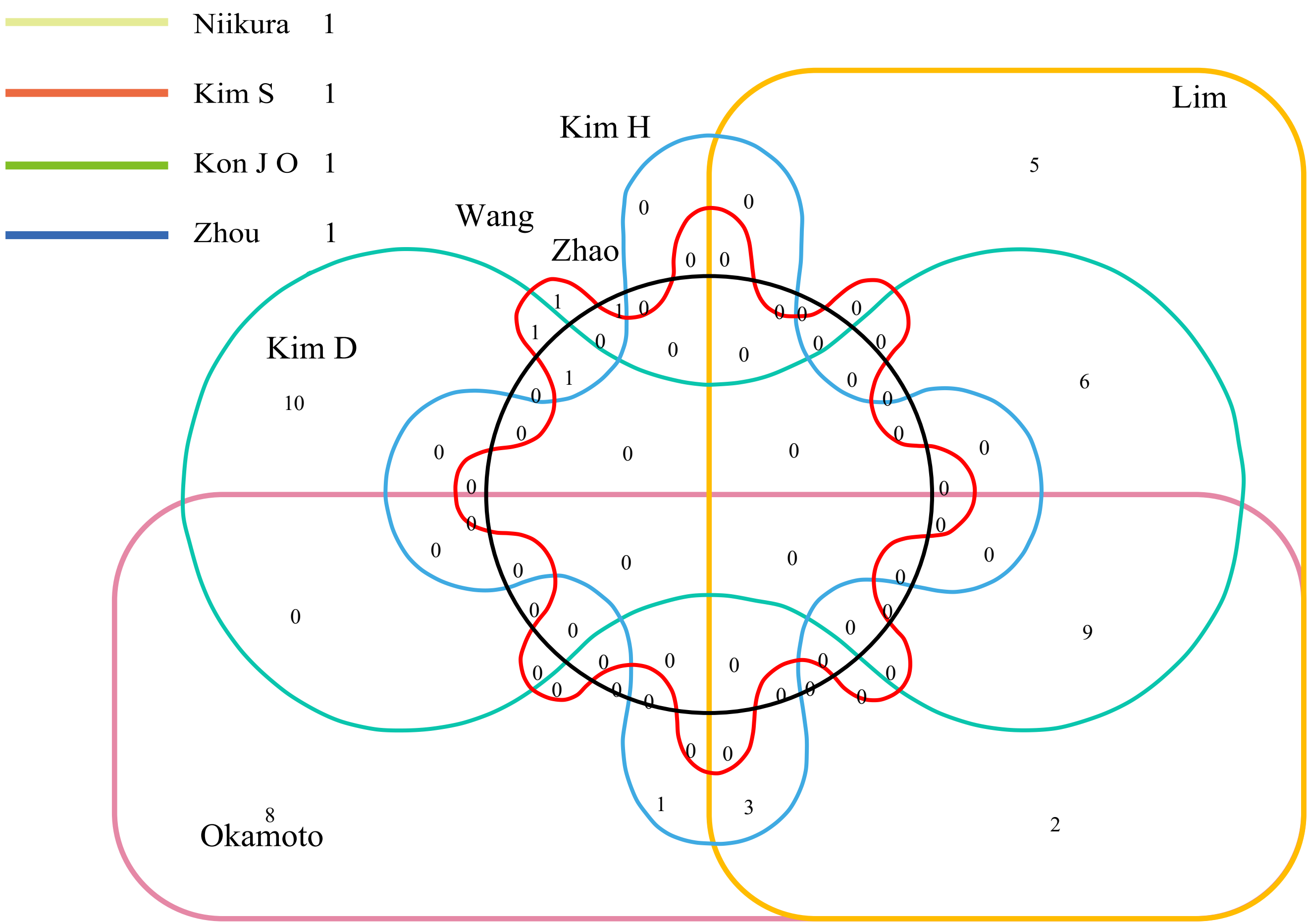
Niikura 1
Kim S 1
Kon J O 1
Zhou 1
Kim H
Lim
Wang
Zhao
Kim D
Okamoto
5
6
9
2
10
0
8
1
3
